# An argument for pandemic risk management using a multidisciplinary One Health approach to governance: an Australian case study

**DOI:** 10.1186/s12992-022-00850-4

**Published:** 2022-07-26

**Authors:** Katie Woolaston, Zoe Nay, Michelle L. Baker, Callum Brockett, Mieghan Bruce, Chris Degeling, Joshua Gilbert, Bethany Jackson, Hope Johnson, Alison Peel, Shafi Sahibzada, Charlotte Oskam, Chad L. Hewitt

**Affiliations:** 1 School of Law, Queensland University of Technology, Brisbane, Australia; 2 CSIRO, Health and Biosecurity Business Unit, Australian Centre for Disease Preparedness, Geelong, Australia; 3 Biosecurity and One Health Research Centre, Harry Butler Institute, Murdoch University, Western Australia, Australia; 4 Australian Centre for Health Engagement Evidence and Values, School of Health and Society, University of Wollongong, New South Wales, Australia; 5 Worimi agriculturalist and researcher, Policy Advisor at the Jumbunna Institute for Indigenous Education and Research, University of Technology Sydney, Australia and PhD Candidate at Charles Sturt University, Bathurst, Australia; 6 Centre for Planetary Health and Food Security, Griffith University, Brisbane, Australia

**Keywords:** One Health, Zoonose, Pandemic, Prevention, Environment, Law, Governance, Australia

## Abstract

The emergence of SARS-CoV-2 and the subsequent COVID-19 pandemic has resulted in significant global impact. However, COVID-19 is just one of several high-impact infectious diseases that emerged from wildlife and are linked to the human relationship with nature. The rate of emergence of new zoonoses (diseases of animal origin) is increasing, driven by human-induced environmental changes that threaten biodiversity on a global scale. This increase is directly linked to environmental drivers including biodiversity loss, climate change and unsustainable resource extraction. Australia is a biodiversity hotspot and is subject to sustained and significant environmental change, increasing the risk of it being a location for pandemic origin. Moreover, the global integration of markets means that consumption trends in Australia contributes to the risk of disease spill-over in our regional neighbours in Asia-Pacific, and beyond. Despite the clear causal link between anthropogenic pressures on the environment and increasing pandemic risks, Australia’s response to the COVID-19 pandemic, like most of the world, has centred largely on public health strategies, with a clear focus on reactive management. Yet, the span of expertise and evidence relevant to the governance of pandemic risk management is much wider than public health and epidemiology. It involves animal/wildlife health, biosecurity, conservation sciences, social sciences, behavioural psychology, law, policy and economic analyses to name just a few.

The authors are a team of multidisciplinary practitioners and researchers who have worked together to analyse, synthesise, and harmonise the links between pandemic risk management approaches and issues in different disciplines to provide a holistic overview of current practice, and conclude the need for reform in Australia. We discuss the adoption of a comprehensive and interdisciplinary ‘One Health’ approach to pandemic risk management in Australia. A key goal of the One Health approach is to be proactive in countering threats of emerging infectious diseases and zoonoses through a recognition of the interdependence between human, animal, and environmental health. Developing ways to implement a One Health approach to pandemic prevention would not only reduce the risk of future pandemics emerging in or entering Australia, but also provide a model for prevention strategies around the world.

## Background

As the world continues to focus on the response to, and recovery from, the COVID-19 pandemic, groups of researchers are working to prevent the next pandemic. They do this because pandemics are increasing in frequency [1, 2]. Most emerging infectious diseases and pandemics are derived via spill-over at the human-animal-environment interface [1, 3]. There are an estimated 1.7 million currently-undiscovered viruses existing within mammalian and avian hosts, up to 850,000 of which could have the capacity to infect humans [4]. The links between emerging infectious diseases and anthropogenic environmental changes are becoming increasingly accepted [5, 6]. In particular, biodiversity loss, climate change, agricultural intensification and the trade of wildlife have all been linked to various diseases and pandemics because these processes disrupt ecosystems, leading to (a) changes in how wildlife and microbes interact, and (b) increased contact among people, animals and pathogens. These processes lead to greater opportunities for pathogen spill-over (when a pathogen is passed from an animal to a human) and increased pandemic risk [7].

Despite these demonstrable links, policy, political discourse and research regarding responses to pandemics largely remain focused on reacting to immediate threats to public health. Typical public health responses include (but are not limited to) contact tracing, mandatory isolation, increasing hospital capacities, stockpiling, distribution and use of personal protective equipment, increased sanitation and sanitary practices, and rapid design, approval and distribution of vaccines [8]. Pandemic response mechanisms addressing the animal and environmental drivers of pandemics remain largely focused on monitoring and management of known zoonoses. That is to say, the governance of pandemics is focused on reactive pandemic response and not long-term pandemic prevention.

Most pandemic law and policy responses around the world remain tightly focused on monitoring reportable zoonotic diseases in wildlife, detecting spill-over events in production animals and humans, and preventing ongoing transmission among people. Although these are measures worthy of further attention and investment, they are passive and/or reactive and do not take action against the underlying drivers of emerging diseases that pose a pandemic risk. Prevention through environmental protection and conservation would require significant structural changes and financial investment, yet the economic and social costs of prevention are far less than the cost of pandemics [9], as evidenced by the COVID-19 outbreak and other recent pandemics such as HIV/AIDS. Pandemic prevention is also expressly part of the obligations of State parties to the *International Covenant on Economic, Social and Cultural Rights* (ICESCR), under Article 12, detailing the right to health [10]. Further, long term pandemic prevention interventions could, if designed correctly, bring about other social, ethical, environmental, and public health benefits.

The ‘One Health’ approach recognises the interdependence between human, animal, and environmental health and can provide a long-term pandemic prevention framework to instigate the transformative change required to ensure that pandemic risks are minimised [11]. One Health frameworks have been useful in responding to emerging infectious diseases in the past, but have not typically been used for ‘deep prevention’, that is, to instigate policy change on environmental and social issues such as land-use change, agricultural intensification, urbanization, climate change and the wildlife trade.

To address the multiple governance areas related to pandemic risk management, we formed a multidisciplinary research/practice group to analyse, synthesise, and harmonise the links between pandemic risk management approaches and issues in different disciplines to provide a holistic overview of current practice in Australia, and to examine the need for reform in Australian legislation and policy. We undertook initial mapping and synthesis over a series of online facilitated workshops in July–August 2020, with additional stakeholders from Government, non-Governmental organisations (NGOs) and research institutions.

We use our multidisciplinary perspectives to argue for a strengthened environmental dimension of One Health approaches to pandemic risk management by critically analysing existing systems in Australia, highlighting examples for transformative change. We begin by outlining the most critical existing pandemic risks in Australia. We then discuss the potential and the limitations of the One Health approach in pandemic risk management, and survey the state of One Health policy in Australia. We conclude with the argument for a holistic, First Nations-led, interdisciplinary One Health approach in Australia and make several specific recommendations that may help bridge the gap between environmental, animal, and public health in Australia, and provide best-practice One Health policy around the world.

### Pandemic risks in Australia

Australia is not immune to emerging infectious diseases from either domestic or international sources; there are various risks associated with Australia’s wildlife and farm animals, biosecurity and international trade, and the drivers of pathogen spill-over such as environmental degradation. This section provides an overview of those risks and some of the governance areas relevant to addressing them.

Australia’s current pandemic risk management system is largely focused on established (i.e. known) biosecurity risks with pandemic potential. In the context of emerging infectious diseases, definitions of biosecurity can be narrowed to focus on ‘the protection of people, animals, ecological systems and the economy from the emergence, entry, establishment and spread of harmful infectious agents and diseases’ [12]. Biosecurity risks are both known and unknown, meaning that both targeted and non-specific policies and mitigation approaches are required. Known pathogen biosecurity risks within Australia include endemic domestic animal pathogens (e.g. Johne’s disease in cattle and sheep) as well as pathogens that emerge via sporadic spill-over from wildlife reservoirs (e.g. Avian influenza from wild birds or Australian bat lyssavirus or Hendra virus from flying foxes) [13]. Nine zoonotic diseases in Australia are categorised as national notifiable zoonotic diseases, according to the Federal Department of Health [14], and each State and Territory government drafts its own notifiable disease lists, which may also include diseases specific to the jurisdiction [15]. However, there is no formal list that identifies which notifiable or non-notifiable pathogens have pandemic potential, and assessments are instead undertaken on an ad-hoc basis.

Known biosecurity risks also arise from outside of Australia, particularly serious domestic animal diseases such as foot and mouth disease or African Swine Fever. Additionally, migratory animals can act as vectors of pathogens across international boundaries through island hopping (e.g. Torres Strait) and avian flyways. One key factor driving zoonotic risk in Australia is globalisation. For example, Northern Australia is deemed to be at ‘increased risk of infectious diseases’ found in South-East Asia, due to its proximity, high rates of trade and tourism activity, thus providing a gateway to the rest of Australia [16]. Recent biosecurity breaches of canine vector-borne diseases, canine hepatozoonosis and the canine monocytic ehrlichiosis [17, 18], while not explicitly of pandemic risk demonstrate the continuing zoonotic risks associated with companion animals [19]. Unknown biosecurity risks have received limited attention, and there are few policies to actively seek out, identify and assess future potential biosecurity risks from domestic or international sources. For example, spill-over of coronaviruses from bats to humans, either directly or via bridging hosts, has been previously recognised as a major pandemic risk [20]. Despite this, only three studies [21–23] have investigated the presence of coronaviruses in Australian bats, resulting in only a handful of Australia’s 81 bat species being sampled with sufficient depth to assess future coronavirus spill-over risk [23]. Prada and others found that 19% of 543 micro bats sampled in the south-west region of Western Australia tested positive for coronavirus infection, including viral species and strains that had never been found before [24].

There are also pandemic risks related to legal and illicit wildlife trade in Australia. Legal and illegal wildlife trade are common, due to Australia being home to an array of valuable reptilian, amphibian and avian species [25], and its proximity to established wildlife trade routes in South-East Asia [26]. Illegal (smuggled) imports of adults, eggs and seed can present direct pest and weed risks as well as transport numerous diseases [27].

A final source of risks for spill-over of pathogens with pandemic potential is the large animal agricultural production systems in Australia. Australia specialises in the large-scale production of livestock and is one of the world’s largest exporters of beef, lamb, mutton and goat [28]. Australian production systems involve animals that are, at times and for varying durations, confined in large numbers in single sites; increasingly, Australia is moving to confined housing arrangements for animal agriculture [29]. These characteristics increase the risk of animal-to-human virus spill-over [30]. These risks are further exacerbated by declining resources provided by the Australian government for on-farm advice and extension services, including support for veterinarians [31].

Other unknown risks continue to be under-investigated, particularly around changes to Australia’s ecosystems, through land clearing, mismanagement of waterways, bushfires and biodiversity decline [32], which increase contact between humans and animals harbouring pathogens and may increase rates of zoonotic disease spread [33]. Zoonotic emerging infectious disease risk is elevated in forested tropical regions experiencing land-use changes, and in environments of wildlife biodiversity. For example, New South Wales experienced high levels of rainfall following the Australian bushfires, dramatically increasing mosquito abundance and, in turn, increasing rates of Ross River Virus (RRV) [34]. Queensland also has been shown to have a high incidence of zoonotic vector-borne diseases, including RRV, Barmah Forest disease, and zoonotic faecal-oral parasitic diseases [35–38].

Australia continues to rank amongst the worst countries for its deforestation and land-clearing rates [39, 40]. The drivers of land-use change include unsustainable agricultural practices, land clearing and deforestation, the encroachment of urban populations into wildlife habitats, the development of new mining sites, and the consequent changes to the management of traditionally owned or ancestral Indigenous lands (where most Indigenous-owned land is managed by non-Indigenous people or companies for agriculture and/or mining) among others [41, 42]. Approximately 22% of infectious diseases in Australia have been associated with land use and native vegetation change, including Hendra virus [43–45].

The number of spill-overs directly related to climate change is expected to increase as the effects of climate change become more and more evident. Australia is one of the leading countries for greenhouse gas emissions per capita [46]. In Australia’s current policy setting, the Paris Agreement 2030 target will not be achieved and, even if fulfilled, emission rates would remain incompatible with a 2-degree emissions budget [47]. Research indicates that even a 1.5-degree temperature intensification in Australia will increase risks of biodiversity loss, natural disasters and species extinction, as well as cause significant social and economic implications [48]. As it stands, Australia is experiencing increasing temperature levels, decreasing rainfall levels, and, as a result, most severe and frequent weather events including drought and longer fire seasons [49, 50].

Also important is the disconnect between the different policies that address known and unknown risks. Syndromic surveillance and diagnostic exclusion testing are heavily relied upon for known biosecurity risks, with legislation and protocols to follow in the event of a positive detection. Lacking are policies and funding to follow up if an individual animal tests positive, and no clear guidelines on what to do in the event of a negative result, but where an unknown infection is suspected. The recent detection of a novel Hendra virus variant in a horse that died with clinical disease consistent with Hendra virus disease, but tested negative using routine diagnostic assays demonstrates the failing of ‘exclusion testing’ surveillance. We cannot truly advance pandemic preparedness without embedding routine investigation of unexplained causes of mortality in animal populations.

### Transformation through a One Health approach

The ‘One Health’ approach to public health decision-making has been championed as the most appropriate policy framework to transform pandemic policies from response to prevention. One Health has been billed as a transformative framework that has the potential to ensure appropriate policies across the pandemic timeline, from environmental prevention to animal monitoring and public health response. The conceptual framework is centred on the recognition of the interdependence of human, animal and environmental health [51, 52], and stems from a recognition amongst the scientific and medical community that veterinary and medical professions could collaborate for mutual benefit, not just locally and nationally but on a global scale. Usage of the term ‘One Health’ originated in 2003 in response to Severe Acute Respiratory Syndrome (SARS) and the spread of highly pathogenic avian influenza H5N1 [53]. A series of strategic goals were developed in 2004, known as the ‘Manhattan principles,’ by the Wildlife Conservation Society [54], and updated to become the ‘Berlin Principles’ in 2019 (the Berlin Principles include stronger language around required actions and include acknowledgement of the climate crisis) [55]. These goals recognise the links between humans, wildlife, domesticated animals and plants, and all nature, and urge world leaders to take action to develop strong institutions to integrate these areas, eliminate or mitigate ecosystem alterations and associated impacts and enhance capacity for cross-sectoral and transdisciplinary collaborations, amongst others.

Despite these well-formulated principles, there is no one recognised definition of One Health, and so organisations and academics have formulated individual definitions with varying elements and principles [56]. These are often linked to the specific values, principles and interests of the respective organisation. A recent attempt has been made by the post-COVID established One Health High Level Expert Panel (OHHLEP) to consolidate various definitions, and while that consolidated definition has since been supported by the World Health Organization (WHO), The Food and Agriculture Organisation (FAO), the World Organisation for Animal Health (OIE) and the United Nations Environment Programme (UNEP), uptake at the ground level is yet to be seen [57].

The various definitions of One Health do have common themes. The ‘One Health’ approach is referenced as ‘integrated,’ ‘trans-disciplinary,’ ‘multi-sectoral’ and ‘holistic.’ The terms ‘interdependent’ and ‘interconnected’ are used when labelling the connection between multiple sectors and disciplines. Alternatively, when not referred to as an ‘approach’, One Health is described as a ‘paradigm’, ‘strategy’, and ‘concept.’ The terms ‘human’/‘animal’ or ‘human health’/‘animal health’ are present in all definitions, however, while the term ‘environment’ is present in nearly all definitions, it is often mentioned only in relation to the environments in which humans and animals operate. For example, the United States Centre for Disease Control (CDC) recognises the interconnection between people, animals, plants, and ‘their shared environment’ [58]. The OIE acknowledges that human and animal health are interdependent and bound to the ecosystems in which they exist [59]. The FAO iterates working together to tackle health threats to animals, humans, plants, and the environment, while WHO states the approach is critical in the animal, human and environment interface [60, 61]. Both FAO and WHO, for example, situate the environmental threats and health alongside human and animal health and potential threats.

There are strengths and weaknesses in the diversity of definitions of One Health. On the one hand, a lack of clear definition means that One Health may be misinterpreted or narrowly applied. On the other hand, the concept’s flexibility means it has received wide acceptance and adoption both regionally, nationally and internationally and can continue to be shaped into the future to match the quick pace of globalisation, technological progression, scientific understanding, and cultural norms. It has been critiqued for being too anthropocentric [62], neglecting environmental health in practice [63, 64], and universalising western science and knowledge acquisition [65, 66].

**Fig. 1:**
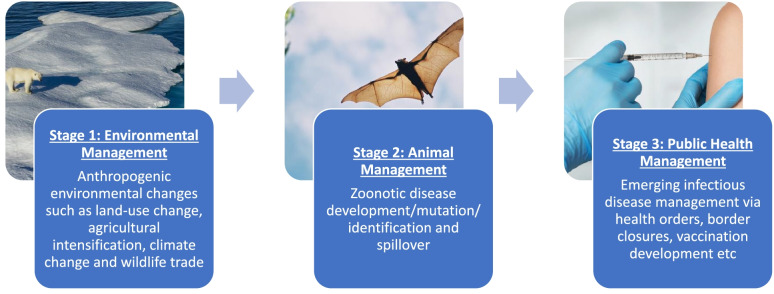
Stages of pandemic risk management

However, the utility of a well-designed and integrated One Health framework is yet to be really tested. We recognise the pros and cons of the various formulations of One Health and consider One Health not as a concept with one inflexible definition, but as a problem-solving framework and value proposition, under which more specific policies are defined and implemented. The framework, and any resulting actions and policies, can be guided by the values and principles arising out of the common definitions, such as the facilitation of open interdisciplinary, multi-sectoral, and cross-cultural strategies. We also acknowledge that One Health can, and should incorporate ‘deep prevention’ policies that focus on and reduce the environmental drivers of pandemics. It can incorporate policies that address the ‘three stages’ of pandemics, as demonstrated in Fig. 1.

Critically for the Australian context, One Health approaches should seek to understand current structural and systemic forces that have threatened the relationship that Indigenous people have to their land and animals. To enable this holistic approach, One Health strategies should be developed and implemented through collaborative and participatory means, with Indigenous knowledges, sciences, and experiences operating alongside and respected by western sciences. Underpinning the efficacy of One Health strategies is the need to secure adequate funding sources with the flexibility to meet identified community needs, collaborative and multidisciplinary research approaches, Aboriginal and Torres Strait Islander workforce and skills base development, and community capacity building, including leadership and self-governance. To this end, researchers have a responsibility to inform themselves about the past and current experiences of Aboriginal and Torres Strait Islander people with research and interventions, to ensure One Health responses in the future support the long-term goal of reconciliation. In the following sections, we describe and analyse the existing One Health policies in Australia, and then make suggestions on its improvement.

### The Australian One Health context

In this section we review the One Health framework as it relates to pandemic prevention and risk mitigation. While the term ‘One Health’ is mentioned in governmental policy documents in just about every State and Territory in Australia, there is no national multidisciplinary One Health body. Individual governmental departments and NGOs are leading One Health policy across the country. For example, the Federal Department of Foreign Affairs and Trade’s Indo-Pacific Centre for Health Security has One Health as a core principle and co-funds research with the Australian Centre for International Agricultural Research on One Health policies across the region. The Federal Department of Agriculture, Water and the Environment, and the Federal Department of Health, has prioritised a One Health approach in their joint ‘One Health Master Action Plan for Australia’s National Antimicrobial Resistance Strategy’ [67]. Wildlife Health Australia, Animal Health Australia, and the Australian Veterinary Association, all integrate One Health into their strategies to some extent. The Commonwealth Scientific and Industrial Research Organisation (CSIRO), is an Australian Government science agency which is developing an Infectious Disease Resilience Mission and an Anti-Microbial Resistance Mission under the guidance of a One Health framework [68].

In Queensland, the Department of Agriculture and Fisheries has a ‘Health and Food Sciences Precinct’, which positions itself as one of the nation’s leading initiatives working towards a One Health approach in health and disease research [69]. There is also a Memorandum of Understanding between Queensland Health, the Department of Agriculture and Fisheries and the Office of Industrial Relations to formalise management standards around an ‘emergency management approach to zoonotic incidents’ [70]. Victoria’s specific approach to Q-fever follows a One Health model that promotes collaboration among multiple stakeholders, including Worksafe Victoria, local governments, the Department of Health and Human Services, Agriculture Victoria and the Chief Veterinary Officer [71, 72]. South Australia Health commits to adopting a One Health approach in its strategic planning documentation [73]. Similarly, Western Australia’s Department of Primary Industry and Regional Development highlights support for a One Health concept through its core biosecurity activities [74].

These examples demonstrate that Australia is relatively advanced when it comes to acceptance of, and referral to the One Health approach. Collaborative initiatives such as the *Australian Antimicrobial Resistance Strategy* and *Hendra Virus Interagency Technical Working Group* are significant attempts to achieve collaboration between human and animal health sectors [75, 76]. Previous research also indicates high levels of support among Australian policymakers and practitioners for a One Health approach to zoonotic disease control and prevention [77, 78]. However, there are several key barriers to implementation that could limit One Health collaboration and its benefits [79].

In the first instance, One Health policies have largely failed to engage with, or even consider, Aboriginal and Torres Strait Islander health, knowledges and connection to Country. First Nations Australians are disproportionately affected by pandemics [80], while simultaneously managing around 40% of Australia’s landmass [81]. One Health and First Nations Lores, including ways of Caring for Country, share many themes. Both see the connections between a healthy environment and the health and well-being of people [82]. The Australian formulation of One Health can, and should, learn from First Nations knowledges and caring practices. First Nations land management practices are associated with improved biosecurity, weed and non-native animal control, general conservation of threatened species, improved fire management and lower greenhouse gas emissions [83].

Second, and like most practical One Health policies, disciplinary fragmentation remains a real and substantial issue. Despite a decade of international and cross-sectoral mobilisation, both One Health advocates and more critical voices remain concerned that all relevant disciplines are not sufficiently engaged in relevant research and policymaking activities, especially experts from social, ecological and environmental health sciences [77]. There is a tendency for public health and biosecurity responses to emerging infectious diseases to focus on controlling cross-border pathogen transfer and community outbreaks, rather than addressing the ecological drivers from which the threats emerge. This fragmentation is exacerbated in Australia by the siloed funding of research activities and a lack of tertiary education about One Health approaches across all three relevant sectors (public health, veterinary sciences and environmental science/policy) [84, 85].

Linked to this fragmentation is a lack of evidence about how different sectors understand One Health, their roles and responsibilities and how they pursue their priorities. More broadly, it is unclear in Australia whether One Health is to be operationalised to reach a holistic understanding of emerging threats or a road map for effective cross-sectoral responses. Discussion of barriers and enablers of One Health cross-sectoral collaboration and policy implementation are rare. Experience suggests that attempts to promote a cross-sectoral approach rarely move beyond rhetoric, even when driven by the best intentions and supported by substantial resources. Arguments tend to focus on the likely benefits of collaboration rather than the required action and outcomes. The complexities of emerging infectious disease control and prevention mean that effective pandemic prevention and early response require genuine cross-sectoral integration and re-sectoring of some institutional and professional responsibilities [86], such as joint human/animal health laboratories and a closer collaboration between the environment and health portfolios. Any such efforts are likely to meet with resistance within and across the relevant sectors [78].

Additionally, because capacities and resources to manage pandemics are limited, prioritisation of the goal of pandemic prevention and resource allocation to this effect requires political decisions about who and what is valued, what must be protected and what is dispensable. One Health does not provide mechanisms for resolving stakeholder conflicts, and, at least in Australia, these discussions and associated decisions have been a matter for experts and implicated industries; citizen and public involvement has been limited. By way of example, an examination of the operationalisation of One Health in Australia to manage the risks of antimicrobial resistance (AMR) indicate that this approach is construed by governing bodies as a rationale to assemble and seek consensus and voluntary collaboration among implicated stakeholders [87]. In the UK, One Health approaches to AMR have been operationalised as requiring government leadership to force collaborations between sectors and establishing mechanisms for sectoral accountability. Australia’s AMR strategy has asked for action from non-governmental actors, with few explicit accountability mechanisms or central controls. The reliance of the Australian economy on extractive industries means that larger deliberations, broader political action and social and economic transformation may be required to overcome the effective veto that stakeholders such as the mining and agriculture industries have over any policy change that requires system reforms, a radical re-orientation of their commercial interests, or both.

Finally, there are several legal challenges to implementing a One Health approach in Australia. Laws and policies identifying and managing the drivers of zoonotic spill-over and pandemics have thus far proven inadequate [88]. This is true globally, but also specifically within Australia. This has never been clearer than with the release of the interim review into the status of Australia’s national environmental protection legislation, the *Environmental Protection and Biodiversity Conservation Act* [89]. The ten yearly review, released in October 2020, clearly states that the legislation is ineffective. Further, the proof of its ineffectiveness is in the continued decline of the environment, measured by the very same factors that are linked to pandemic risk:

‘Australia’s natural environment and iconic places are in an overall state of decline and are under increasing threat. The pressures on the environment are significant – including land-use change, habitat loss and degradation, and feral animals and invasive plant species. The impact of climate change on the environment will exacerbate pressures and contribute to further decline. In its current state, the environment is not sufficiently resilient to withstand these threats. The current environmental trajectory is unsustainable.’ [90]

Not only are Australia’s environmental laws failing, but they also fail to make this connection between environmental and public health. In the following section, we identify opportunities for transformation, towards a holistic One Health approach, in Australia’s socio-cultural, political, economic, and regulatory systems.

### Opportunities for transformation

Policymakers should begin efforts to collaborate in new ways to reshape the way Australia prevents and prepares for pandemics. We recommend three focus areas: 1. A coordinated and well-supported ‘One Health’ system that is integrated and Indigenous-led; 2. Research, education and training funding and support; and 3. Inclusion of networked policies and laws that consider pandemic risk.

#### A coordinated and well-supported ‘One Health’ system

Preventing pandemics by managing environmental drivers – such as climate change, land-use change, and biodiversity loss – requires stakeholder engagement across all sectors and scales, in all communities. Australia’s One Health should be both coordinated, across governance scales and disciplinary siloes, and collaborative with diverse stakeholders. This includes wide coordination of policy spheres such as biosecurity, trade, agriculture, private land management, environmental and wildlife management, as well as stakeholder engagement with Indigenous peoples, farmers, rangers, ecologists, and community members, among many others.

One mechanism to enable such a coordinated and collaborative One Health system is the creation of a new One Health governance body within the Federal Government system, designed and steered by a cross-sectoral and transdisciplinary group. This recommendation is endorsed by the CSIRO Biosecurity Report, which noted the need for connective governance that included digitizing and data sharing between jurisdictions and sectors, strong stakeholder engagement, streamlining domestic trade, strengthening international relationships and improving supply chain risks [91]. We would add that such a body should sit across all departments, and so be housed in the Office of the Prime Minister and Cabinet. A nationally coordinated governance body should seek to connect the dots between existing domestic initiatives and policies by ensuring the inclusion of deep prevention research and policy.

As no one group can create a healthy country without institutional support, Australia’s One Health governance system must be collaborative. A shared responsibility approach recognises the need for coordinated pandemic prevention, preparedness, detection, response and recovery [92], through collaboration across jurisdictions, disciplines, and community groups [90]. The creation of a centralised One Health governance body is in no way antithetical to a shared, collaborative governance scenario. Rather, a centralised governance mechanism can provide the institutional stability needed for collaborative and bottom-up mechanisms to thrive [93]. Effecting a shared responsibility approach to One Health governance requires a common understanding of priorities, values, and roles throughout the socio-ecological system [94]. In Australia, a shared responsibility approach to One Health governance should be congruent with the ‘Healthy Country, Healthy People’ policy and First Nations laws [95].

An Aboriginal and Torres Strait Islander-led One Health policy requires self-determining structures, upskilling and collaboration with Indigenous Peoples. This includes local voices to co-create policies, laws, programs and services, with a strong partner and feedback processes and devolution of power to the local level [96]. A ‘Healthy Country, Healthy People’ approach to One Health would also give priority to more-than-human health as a means of pandemic prevention, including the wellbeing of animals and ecosystems [97].

#### Research and education funding and support

The current state of limited coordination and prioritisation of Australia’s One Health efforts has led to gaps in Australia’s understanding of national risks and drivers regarding spill-over and pandemics. Looking forward, research priorities need to include, but are not limited to: identifying endemic and introduced zoonotic risks in Australian wildlife, livestock and companion animals, as well as invasive species; One Health/Healthy Country research with an emphasis on Indigenous knowledges; and collaborations with Indo-Pacific and wider neighbours to underpin regional resilience.

Building an effective network of One Health stakeholders and policies in Australia requires insights across the natural and social sciences, Indigenous and local knowledges, business and economics, humanities and the arts, and many other diverse experiences and sources of expertise.

A non-exhaustive list of relevant research includes:

- Natural sciences including whether genome mapping can be used to predict spill-over risk and pandemic potential and the role of viral discovery research in spill-over risk;
- Data and technological development including improvements in data mapping.
- Geography and policy including investigations into how to improve key areas of law and policy that intersect with zoonotic spill-over (e.g. wildlife trade, agricultural intensification, land-use change, climate change, natural disaster etc.) and what kinds of interventions outside of direct management of risk could be formulated, for instance, regulations that promote sustainable diets.
- Justice and equity including deliberation into the role of the Australian government in addressing increased spill-over risk in low-income countries and how to build an ‘infrastructure of trust’ to increase community respect of government and scientists?

#### Networked policies and laws

Given the complex and multisectoral nature of zoonotic risks, and the interconnectedness of global players, it is unlikely that a single institution or instrument could adequately prevent the ‘era of pandemics’ [98]. Top-down regulation – generally referring to legislation or otherwise binding legal obligations – rarely mirrors the dynamism and complexity of environmental challenges, especially those that scale across ecosystems and jurisdictions [99]. Equally, bottom-up policies – such as self-regulation, voluntary initiatives, or education programmes – can result in the fragmentation of global environmental challenges, and lack longevity if not institutionally supported.

The network of existing environmental law regimes should be harnessed and enhanced to take a systems-thinking approach to pandemic prevention. Australia’s One Health governance system should incorporate and exceed relevant international standards, applied in a way that best addresses the risks and opportunities in Australia. Relevant environmental standards for pandemic prevention include, but are not limited to, those in the Convention on Biological Diversity, the United Nations Framework Convention on Climate Change, the Convention on the International Trade in Endangered Species of Wild Fauna and Flora, the United Nations Convention to Combat Desertification and the Ramsar Convention on Wetlands of International Importance Especially as Waterfowl Habitat. Critically, the advancement of environmental law in Australia needs to be meaningfully guided by Aboriginal and Torres Strait Islander engagement and participation. As a starting point, Australia could outline a series of Healthy Country, Healthy People and One Health targets that are similar to the United Nations’ Sustainable Development Goals. National standards should also adopt a whole of country management perspective, including the integration of human, animal and environmental health priorities.

Several actions are necessary on a more immediate and localised scale to implement a One Health system in Australia. First, there is a need for a centrally coordinated, systematic and ongoing surveillance system, with concurrent human, animal and environmental research, to better understand emerging infectious disease threats [100]. Second, One Health should be incorporated into land-use and development planning documents, such as Environmental Impact Assessments (EIAs), in a way that is respectful of, and responsive to, the perspectives of Indigenous communities and landholders that may be affected by the proposed project [96. This would necessarily involve recognising the broader landscape and environmental impacts across lands. Third, various options exist for reducing the number of animals being used in production systems. These include regulatory interventions that: enable transitions from high-meat diets, [101] increase on-farm advice and extension focused on diversifying farms, convert farmland into conservation sites, incentivise on-farm conservation actions and restrict approvals of concentrated feeding lots [102].

Reducing the amount of time animals spend in confined circumstances and improving the use of inputs on farms carry benefits for preventing pandemics and improved animal welfare. Australians are increasingly concerned with the welfare of animals on farms [103]. Focusing on improving animal welfare standards and enforcement in Australia could, therefore, help prevent pandemics. One of the key ways in which to improve animal welfare standards and enforcement in Australia is to establish, at state and territory and Federal levels, independent regulatory bodies with powers to set and effectively enforce animal welfare standards and to administer the relevant Animal Welfare Acts using existing and emerging methods [104].

Finally, promoting this network of One Health laws and policies, from the local to the global scale, will require economic support. Government funding requires redirection and realignment towards One Health/Healthy Country priorities. Research demonstrated that redirecting government expenditure towards Indigenous-led initiatives can lead to economically viable, culturally appropriate, *country*-based, sustainable solutions that enhance peoples’ wellbeing, as well as having benefits for natural resource management [105]. Further, economic incentives should be redirected away from subsidies for intensive agricultural and industrial overfishing, towards incentivising positive action for biodiversity management, such as species or ecosystem restoration [106].

## Conclusions

Pandemics are becoming more frequent and more severe, and are a threat to the wellbeing of every Australian. The current pandemic strategy depends on responding to spill-over events after they occur with public health measures and technological responses. However, the COVID-19 pandemic has demonstrated that such response actions are not comprehensive nor fast enough to avoid global disruption and harm.

Further, meaningful risk management strategies go much deeper than monitoring animal diseases and reducing their spread once spill-over has occurred. Pandemic and zoonotic spill-over risk management frameworks are multi-faceted, complex concepts that enter into many spheres of governance, including land clearing and habitat destruction, poverty, natural and cultural rights, trade, tourism, development, animal welfare, disease control, agribusiness, resource extraction, and public health. All these areas must be factored into a governance paradigm to effectively address prevention and risk management. The scope of this task is not amenable to any one group or disciplinary practice area alone. One Health can, and should, provide the basis for consolidating expertise and knowledges to formulate direct policies across the prevention spectrum.

In this article we have sought to demonstrate the current state of pandemic risk management policies in Australia, the need for improvement, and strategies to start to reshape the way that Australian governance systems define, prevent and prepare for pandemics. Australian leaders can and should strive for healthy and resilient communities and landscapes by taking steps to further understand and evidence the linkages between environmental change and emerging diseases in Australia and building new communities of expertise. Most importantly, Australian leaders should prioritise the development of an integrated, highly sensitive and holistic One Health/Healthy County system and become a world leader in pandemic risk management.

## Data Availability

No applicable.

## Abbreviations

AMR: Antimicrobial resistance
CDC: United States Centre for Disease Control
CSIRO: Commonwealth Scientific and Industrial Research Organisation
EIAs: Environmental Impact Assessments
FAO: Food and Agriculture Organization
ICESCR: International Covenant on Economic, Social and Cultural Rights
NGOs: Non-Governmental organisations
OHHLEP: One Health High Level Expert Panel
OIE: World Organisation for Animal Health
RRV: Ross River Virus
SARS: Severe Acute Respiratory Syndrome
UNEP: United Nations Environment Programme
WHO: World Health Organization
